# Efficient [Fe-Imidazole@SiO_2_] Nanohybrids for Catalytic H_2_ Production from Formic Acid

**DOI:** 10.3390/nano13101670

**Published:** 2023-05-18

**Authors:** Christos Gkatziouras, Maria Solakidou, Maria Louloudi

**Affiliations:** Laboratory of Biomimetic Catalysis & Hybrid Materials, Department of Chemistry, University of Ioannina, 45110 Ioannina, Greece; ch.gkatziouras@uoi.gr (C.G.); m.solakidou@uoi.gr (M.S.)

**Keywords:** formic acid, hydrogen production, dehydrogenation of formic acid, iron nanocatalysts, imidazole, pyridine

## Abstract

Three imidazole-based hybrid materials, coded as IGOPS, IPS and impyridine@SiO_2_ nanohybrids, were prepared via the covalent immobilization of N-ligands onto a mesoporous nano-SiO_2_ matrix for H_2_ generation from formic acid (FA). BET and HRTEM demonstrated that the immobilization of the imidazole derivative onto SiO_2_ has a significant effect on the SSA, average pore volume, and particle size distribution. In the context of FA dehydrogenation, their catalytic activity (TONs, TOFs), stability, and reusability were assessed. Additionally, the homologous homogeneous counterparts were evaluated for comparison purposes. Mapping the redox potential of solution E_h_ vs. SHE revealed that poly-phosphine PP_3_ plays an essential role in FA dehydrogenation. On the basis of performance and stability, [Fe^2+^/IGOPS/PP_3_] demonstrated superior activity compared to other heterogeneous catalysts, producing 9.82 L of gases (VH_2_ + CO_2_) with TONs = 31,778, albeit with low recyclability. In contrast, [Fe^2+^/IPS/PP_3_] showed the highest stability, retaining considerable performance after three consecutive uses. With VH_2_ + CO_2_ = 7.8 L, [Fe^2+^/impyridine@SiO_2_/PP_3_] activity decreased, and it was no longer recyclable. However, the homogeneous equivalent of [Fe^2+^/impyridine/PP_3_] was completely inactive. Raman, FT/IR, and UV/Vis spectroscopy demonstrated that the reduced recyclability of [Fe^2+^/IGOPS/PP_3_] and [Fe^2+^/impyridine@SiO_2_/PP_3_] nanohybrids is due to the reductive cleavage of their C-O-C bonds during catalysis. An alternative grafting procedure is proposed, applying here to the grafting of IPS, resulting in its higher stability. The accumulation of water derived from substrate’s feeding causes the inhibition of catalysis. In the case of [Fe^2+^-imidazole@SiO_2_] nanohybrids, simple washing and drying result in their re-activation, overcoming the water inhibition. Thus, the low-cost imidazole-based nanohybrids IGOPS and IPS are capable of forming [Fe^2+^/IGOPS/PP_3_] and [Fe^2+^/IPS/PP_3_] heterogeneous catalytic systems with high stability and performance for FA dehydrogenation.

## 1. Introduction

The clean energy potential of molecular hydrogen (H_2_) has garnered significant interest due to its favorable characteristics, such as its energy density, which is 2.6 times greater than that of gasoline, and the absence of toxic byproducts during the combustion process [1,2,3]. However, free H_2_ does not exist on Earth and a primary energy source is required for its production. Within the concept of a cyclic economy, the production of H_2_ that is fully reliant on renewable sources includes two independent processes. The first process involves the generation of H_2_ through the dehydrogenation of a hydrocarbon substrate, while the second one involves the reduction of CO_2_ to produce hydrocarbon fuels [4,5]. The technology in question has the potential to revolutionize the industry, as it is worth noting that a significant majority of H_2_ generation, specifically 96%, currently relies on non-renewable sources such as fossil fuels [6]. Formic acid (FA) is a highly promising substrate for providing H_2_, owing to its favorable cost and simplicity of handling [7,8]. The decomposition of FA occurs via two possible pathways; it is imperative to avoid Reaction (1), as the produced CO is detrimental to the functionality of fuel cells due to its toxic nature. Equation (2) exhibits a thermodynamically allowed reaction pathway, as evidenced by a negative Gibbs free energy change of −32.9 kJ/mol at elevated temperatures. However, the reaction is kinetically blocked, necessitating the use of a catalyst in order to accelerate the process [9];

HCOOH (l) → H_2_O (l) + CO (g) ΔG_o_ = −12.4 kJ/mol (1)

HCOOH (l) → H_2_ (g) + CO_2_ (g) ΔG_o_ = −32.9 kJ/mol (2)

Since 1967, with the first reported catalytic system for FA dehydrogenation [10], numerous studies have been conducted to identify highly effective homogeneous and heterogeneous catalysts that can selectively produce H_2_ and CO_2_ from FA under mild conditions. Complexes of Ir [11,12,13], Ru [14,15,16,17,18,19] and Rh [20] have been extensively investigated as noteworthy catalysts. Beyond the nature of the metal, the electronic and steric properties of the organic ligand play a crucial role in determining catalysts’ reactivity and regulating metal–substrate interactions during catalysis [8]. Within this context, N,N′ bidentate ligands, including *imidazole* and *pyridyl* groups, have been proved to be very effective due to the nitrogen-atom donor capacity of the ligand towards the metal center [21]. In several studies conducted by the research group of Himeda [11], refs. [22,23,24] on Ir complexes using different N,N’ bidentate ligands (*imidazole* and *pyridyl* moieties), the notable catalytic activity and stability of these substances have been demonstrated. In addition to the nitrogen donor ligands, imidazolium-based ionic liquids (ILs) have been observed functioning as effective reaction media by aiding in the stabilization of various transition metal catalysts and supporting the catalyst’s recyclability [25]. The reversible decomposition of FA into CO_2_ and H_2_ in the ionic liquid (IL) 1,3-dipropyl-2-methylimidazolium formate was investigated by Yasaka et al. in 2010 [26]. In their study, the research group of Deng [16] utilized the commercially accessible IL 1-butyl-3-methylimidazolium chloride (BMimCl) as a solvent for the decomposition of FA, employing the Ru-based catalyst, [{RuCl_2_(p-cymene)}_2_], with iPr_2_NEt/HCOONa as a base. The experimental setup yielded 725 mL of gas within a 2 h timeframe, resulting in a TON_2h_ value of 240. Berger et al. [27] reported a catalytic system consisting of RuCl_3_ dissolved in 1-ethyl-2,3-dimethylimidazolium acetate ionic liquid (IL) as the solvent. The resulting catalyst (RuCl_3_/[EMMIM][OAc]) achieved a turnover frequency (TOF) of 150 h^−1^ at 80 °C, and it was able to undergo recycling for up to 10 cycles [27]. Even if a multitude of research studies unequivocally indicate that the utilization of ionic liquids (ILs), bearing imidazolium moieties, exhibit exceptional properties as reaction media [25], the time-consuming synthesis process and high cost of ILs limit their use [28].

Most catalytic systems which exhibit high efficiency in producing H_2_ consist of metal centers of noble metals that are both scarce and costly. However, the scientific community has begun synthesizing catalysts utilizing non-noble transition metals due to their cost-effectiveness, non-toxicity, and abundance. First-row transition metals, such as iron (Fe) [29], cobalt (Co) [30], and nickel (Ni) [31], that possess diverse σ-donor ligands have effectively catalyzed the process of FA dehydrogenation, which was previously restricted to precious metals [32,33,34]. In more recent studies, Beller et al. synthesized the non-precious Mn(pyridine-imidazoline)(CO)_3_Br complex for FA dehydrogenation, producing 14 L of H_2_ + CO_2_ within 3 days. Although the activity was satisfied, the complex produced more than 2500 ppm of poisonous CO [35].

Despite the good catalytic performance of the homogeneous molecular systems, they exhibit a deficiency in their capacity for recycling, which can be overcome by grafting the catalytic metal complexes onto a solid matrix [36]. The properties required for catalyst supports include chemical stability, a high specific surface area, and the ability to disperse molecular unities on their surface. For reference, porous silicas exhibit a significant portion of the aforementioned properties such as high pore size and specific surface area [37]. Furthermore, silica can be easily manipulated through the modification of its synthetic parameters such as temperature, reaction time, and amount of silica source, via the modification of the calcination condition [38]. To date, there have been a limited number of immobilized homogeneous metal catalysts utilized for FA dehydrogenation. For instance, the research group of Laurenczy [39] immobilized a Ru-phosphine homogeneous catalyst onto various materials such as resin, polymer, and zeolites through ion exchange, coordination, or absorption, albeit with unsatisfactory catalytic performance. Manaka et al. [40] found that the immobilized [Cp*Ir(pyridylimidazoline)(H_2_O)]@SiO_2_ has the same activation energy E_a_ in comparison with its homogeneous counterpart. However, the reduction in collision frequency resulted in a decrease in reaction velocity, with the authors stating that efficient agitation control is necessary in order to achieve the implementation of the immobilized complex in future H_2_ technology [40]. In a recent study, the utilization of a hybrid catalyst [Ir_PicaSi_SiO_2_] comprising the Cp*Ir(R-pica)X complex which was immobilized onto mesoporous silica was examined, showing satisfactory activity but low stability [41]. The best to our knowledge, our laboratory was the first to have immobilized the non-precious complexes Fe^2+^-RPPh_2_ and Fe^2+^-polyRPhphos onto a mesoporous SiO_2_ surface. Fe^2+^/RPPh_2_@SiO_2_ has the remarkable ability to produce a maximum of 14 L of H_2_ within 6 h, whereas the homogeneous Fe^2+^/RPPh_2_ was completely inactive [42]. 

In this study, we present three imidazole-based hybrid materials, namely IGOPS, IPS and impyridine@SiO_2_ nanohybrids, prepared by means of the covalent immobilization of N-ligands onto a mesoporous nano-SiO_2_ matrix. We show hereafter that their integration with the low-cost Fe^2+^ in combination with a polydentate alkyl-phenyl-phosphine ligand (PP_3_) produces efficient and reusable heterogeneous catalytic systems for H_2_ production from formic acid. The role of PP_3_ phosphine in catalysis is investigated and discussed. IGOPS was the best among the nanohybrids, which contributed to the formation of 8.42 L of gasses (H_2_ + CO_2_) within 4 h, while IPS showed remarkable stability. The catalytic drop efficiency was investigated, and it was attributed to (i) the accumulation of H_2_O, derived from the FA stock which contains 2.5% water. We demonstrate herein that the catalysis’ inhibition by H_2_O is reversible, and it can be overcome by a straightforward washing and drying procedure of [Fe^2+^-imidazole@SiO_2_] nanohybrids; (ii) the reductive cleavage which occurred during catalysis of the C-O-C bond of IGOPS and impyridine@SiO_2_ nanohybrids. An alternative grafting procedure is suggested to avoid the fragile C-O-C bond; this was applied for the IPS nanohybrid where the C-O-C group was replaced by an aliphatic C-C-C, resulting in the high durability of IPS in catalysis. Overall, we demonstrate here that the use of nanohybrids in conjunction with non-noble metals such as Fe^2+^ in FA dehydrogenation catalysis for H_2_ production has a high potential, offering flexible, convenient, and low-cost alternatives.

## 2. Materials and Methods

### 2.1. Materials

FA (formic acid) (97.5/2.5 H_2_O [*v*/*v*]), [Fe(BF_4_)_2_6H_2_O] and PP_3_ (P(CH_2_CH_2_PPh_2_)_3_) (98% purity) were acquired from Sigma Aldrich, 3050 Spruce St Saint Louis, MO, 63103-2530 USA, and kept under argon, while Merck, 26 East Lincoln Avenue, P.O. Box 2000, Rahway, NJ 07065 USA, supplied the solvent propylene carbonate. Our earlier publication detailed the synthetic pathway of IGOPS and IPS nanohybrids [43,44]. Details about the synthetic procedure of impyridine@SiO_2_ are provided in the Supplementary Material (Figure S1). TGA, FT-IR and Raman measurements confirmed the successful synthesis of nanomaterials and were provided afterwards.

### 2.2. Characterization Techniques

A Nicolet IS5 system equipped with OMNIC FTIR Software 9.2.86 was used to acquire FT/IR spectra in the range of 4000 to 400 cm^−1^ with a resolution of 2 cm^−1^ and 100 scans. Raman spectra were recorded using a Raman HORIBA-Xplora Plus spectrometer connected to an Olympus BX41 microscope. As an excitation source, a 785 nm diode laser was employed, and the laser beam was focused on the sample using a microscope. Before measurement, each powder material was formed into a pellet by gently pressing it between two glass plates. We employed a 15 mW laser and discovered via trial and error that at this low intensity, the crystal phase stayed unaltered. Typically, Raman spectra with a reasonable signal-to-noise ratio were collected for 15 accumulations at 30 s.

The monitoring of Fe^2+^ species detected in the solution after the end of the catalytic reaction was realized using a Lamda 35 Perkin Elmer UV/Vis spectrometer. Thermogravimetric analysis (TGA-DTA) was performed using a SETARAM TGA 92 analyzer with a heat rate of 10 °C/min from 25 °C to 800 °C and a flow rate of 20 mL/min for the oxygen carrier gas. The organic loading of imidazolium in IGOPS was 0.82 mmol/g, in IPS@SiO_2_ it was 0.45 mmol/g, while the impyridine loading of impyridine@SiO_2_ nanoparticle was 0.24 mmol/g. The measurement of the specific surface area (SSA) and pore size of the nanomaterials was conducted using a Quantachrome NOVAtouch_LX2. This involved recording the N_2_ adsorption–desorption isotherms at a temperature of 77 K. The specific surface area (SSA) was determined by utilizing the absorption data points within the 0.1–0.3 range of relative pressure (P/Po). The analysis of the pore radius was conducted using the BJH method [45] within a range of 0.35–0.99 P/Po. The morphology of the nanomaterial was examined through the utilization of high-resolution transmission electron microscopy (HRTEM) with a Philips CM 20 microscope that was operated at 200 kV, offering a resolution of 0.25 nm. Prior to conducting measurements, the samples went through a mild grinding process using a mortar and were subsequently loaded in a dry state onto a support film composed of Lacey Carbon with a mesh size of 300 (Cu). The images that were recorded were subjected to analysis using the Gatan Digital Micrograph 3.9 software. 

### 2.3. Catalytic Experiments

At a temperature of 80 °C (±1 °C), catalytic reactions were conducted in a double-walled thermostated reactor with the addition of Ar gas and constant stirring. The reactor was connected online to a GC system (Shimadzu GC-2014 Gas Chromatograph with Thermoconductive Detector, GC-TCD, equipped with a Carboxen-1000 column) for the analysis and qualification of produced gases, while the total volume of evolved gases was measured with a manual gas burette. In a typical catalytic experiment, 7.5 μmol of Fe(BF_4_)_2_6H_2_O and 15 μmol of IGOPS or IPS or impyridine@SiO_2_ were added to a 7 mL propylene carbonate/FA mixture (5 mL/2 mL). After 10 min of vigorous stirring, 7.5 mol of PP_3_ was introduced to the reaction. For the calculation of TONs and TOFs, the procedure described in [17,42] was followed (Supplementary Material). The redox potential (E_h_) was measured using a Metrohm platinum electrode (type 6.0401.100) versus a standard hydrogen electrode (SHE) that had been calibrated with a Ferri/Ferro solution.

*Continuous operation system*: Upon the consumption of approximately half of the FA (1 mL), resulting in the production of 1200 mL of gasses (H_2_ + CO_2_), an additional 1 mL of FA was introduced. This process was repeated as soon as 1200 mL of gases were produced, until the reaction stopped. In this way, the catalyst’s performance was believed to be optimized by avoiding the imposition of extreme pH changes.

*Recycling experiments*: When catalytic gas evolution stopped, the solid catalyst was collected by centrifugation (4000× *g*, 15 min), washed with 8 mL methanol and dried overnight at 100 °C. The collected solid was added for a second use under the same catalytic conditions (continuous operation system), with no further Fe^2+^ or [imidazole derivative@SiO_2_] nanohybrid addition. This procedure was repeated until the reaction stopped.

## 3. Results and Discussion

### 3.1. Characterization of Hybrid Materials

**Figure 1:**
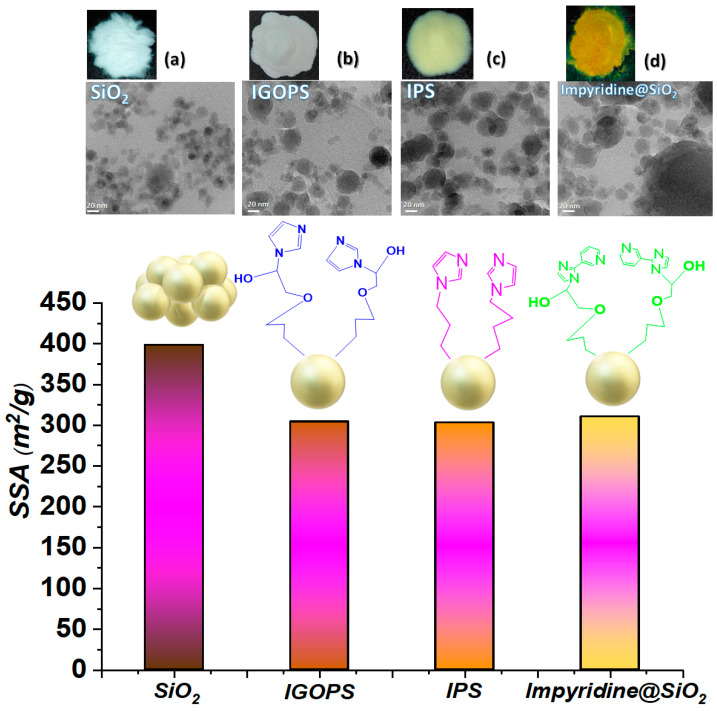
Photos, TEM images and SSA of functionalized nano-SiO_2_ hybrid materials. (**a**) SiO_2_; (**b**) IGOPS; (**c**) IPS; (**d**) impyridine@SiO_2_.

Figure 1 depicts the surface functionalization of SiO_2_ NPs, as shown by the color changes of the white-pristine SiO_2_ particles to yellow and orange-brown. The BET results (Figure 1) demonstrate that grafting reduces the specific surface area by 25%, accompanied by a reduction in pore diameters; for further information, see Figures S3 and S4 in the Supplementary Material File and Table 1. Figure 1’s top row depicts TEM images of SiO_2_ and functionalized SiO_2_-imidazole derivative hybrids. SiO_2_ nanoparticles (Figure 1a, upper left) have a spherical shape, mixing to create chain-like agglomerates, while the average size distribution is 25 nm, and we noticed an increase when the immobilization of the imidazole derivative occurred (e.g., Size_Impyridine@SiO2_ = 35 nm, Figure S5a–d). As a result of the grafting, the accessible pores were filled (the average pore volume decreased from 0.71 cc g^−1^ for SiO_2_ to 0.41, 0.45, 0.59 cc g^−1^ for IGOPS, IPS and impyridine@SiO_2_, respectively), making the modified surface area more compact (Figure 1b–d).

**Figure 2:**
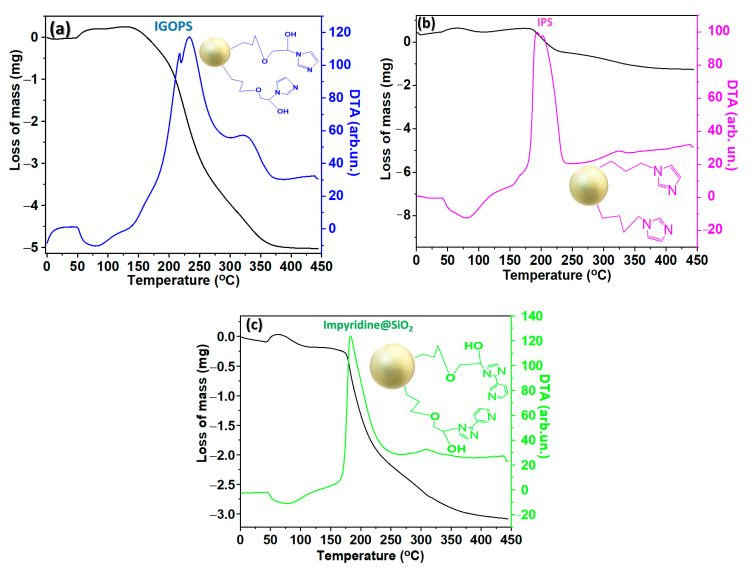
Thermographs of SiO_2_-imidazole derivatives. (**a**) IGOPS; (**b**) IPS; (**c**) impyridine@SiO_2_.

Thermogravimetry of nanohybrid SiO_2_-imidazole derivatives show increasing mass loss, accompanied by exothermic–endothermic curves in all cases. The exothermic changes are due to the combustion of the organic groups, while the endothermic ones are due to the presence of organic solvents that may be present in the sample. More specifically, the IGOPS nanohybrid (Figure 2a) provides a wide exothermic curve in the range of 250–450 °C, with a maximum at 380 °C, which corresponds to the weight loss of imidazole groups on the SiO_2_ surface. Organic loading is 14% corresponding to 0.82 mmol of imidazole/g of SiO_2_. The endothermic curve at a temperature of 50 °C corresponds to the presence of the organic solvent, and it is not included in the calculation of the organic loading (see Table 1). In a same way, the organic loading of IPS and impyridine@SiO_2_ is equal to 5% and 6% (range of the peak, 250–350 °C, with a maximum at 300 and 290 °C for IPS and impyridine@SiO_2_, respectively), corresponding to 0.45 and 0.24 mmol organic ligand/g of the modified material, respectively.

**Figure 3:**
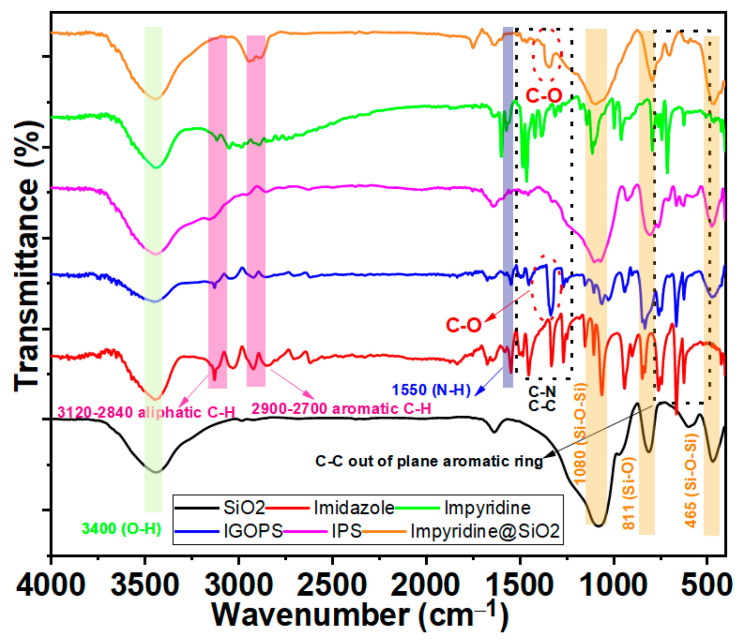
FT-IR spectra of SiO_2_ (**black line**), imidazole (**red line**), IGOPS (**blue line**), IPS (**pink line**), impyridine (**green line**) and impyridine@SiO_2_ (**brown line**).

Figure 3 depicts the FTIR spectra of the hybrid materials, IGOPS, IPS, and impyridine@SiO_2_, as compared to nonfunctionalized SiO_2_ and powders of free imidazole and impyridine. SiO_2_ is defined (black line) by the 465, 811, and 1080 cm^−1^ peaks, which may be attributed to the Si-O-Si and Si-O bond’s asymmetric stretching vibrations, respectively [46]. Imidazole and impyridine are accompanied by the characterizing bend vibration of the N-H bond at 1550 cm^−1^ [47]. The peaks at the regions of 3120–2840 cm^−1^ and 2900–2700 cm^−1^ are attributed to the stretching vibrations of aliphatic and aromatic C-H bonds [47]. Stretching vibration modes of C-C and C-N bonds of imidazole rings appeared in the regions of 1500–1400 cm^−1^ and 1335–1250 cm^−1^, respectively [48]. The FTIR spectra of IGOPS, IPS, and impyridine@SiO_2_ hybrid materials are characterized by the asymmetric stretching vibrations of Si-O-Si and Si-O bonds (1075, 460 cm^−1^ and 805 cm^−1^) derived from the silica support [49]. The downward shift, i.e., −5 cm^−1^, of those bonds suggests the vibrational interaction of nano-SiO_2_ with imidazole functionalities [44]. In the case of IGOPS and impyridine@SiO_2_ hybrids, the appearance of a band at 1320 cm^−1^ is indicative of the C-O stretching bond which they have in their molecular structure (see the Supplementary Material, Figure S2). In the FT-IR spectra of all hybrid materials, the bands observed in the regions of 1500–1400 cm^−1^ and 1335–1250 cm^−1^ are assigned to C-C and C-N bonds of imidazole rings [43]. Interestingly, these bands of IGOPS are more intense in comparison with those of the IPS and impyridine@SiO_2_ nanohybrids; this is due to the higher organic loading of IGOPS of 14% vs. 5% and 6% for IPS and impyridine@SiO_2_, respectively. Overall, the current FTIR measurements indicate the covalent attachment of imidazole and impyridine compounds onto the SiO_2_ surface of IGOPS, IPS and impyridine@SiO_2_ nanohybrids. 

**Figure 4:**
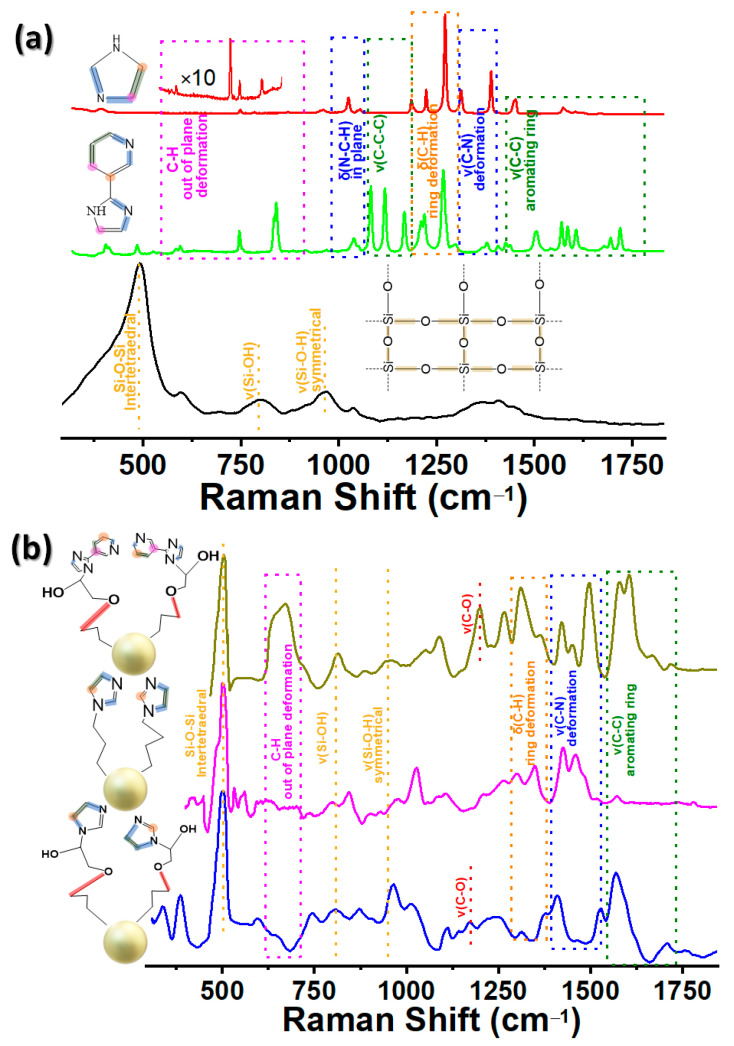
Raman spectra of (**a**) SiO_2_ (black line), imidazole (red line), impyridine (green line) (**b**) IGOPS (blue line), IPS (pink line), and impyridine@SiO_2_ (brown line).

In addition to FT-IR, Raman spectroscopy is a very sensitive method used to study the internal siloxane configurations and surface silanol groups of the silica supporting nanomaterial. However, it is possible to detect the distinctive vibrations of imidazole and impyridine. The Raman spectra of the pristine compounds, as well as the IGOPS, IPS and impyridine@SiO_2_ nanohybrids, are depicted in Figure 4a,b. As we can see, imidazole and impyridine organic ligands show numerous peaks, assigned to the out-of-plane and ring deformation of different vibrations such as v(C-H) (600–850 cm^−1^), δ(N-C-H), (982–1084 cm^−1^), v(C-C-C) (1073–1189 cm^−1^), δ(C-H) (1090–1308 cm^−1^), v(C-Ν) (1310–1404 cm^−1^), and v(C-C) (1429–1781 cm^−1^) [50]. Vibrations of the silica matrix, namely siloxane (Si-O-Si) intertetrahedral modes, are observable in the 300–600 cm^−1^ area [18]. Moreover, the silica matrix shows a characteristic band at ∼800 cm^−1^ attributed to the symmetric stretching vibration of (Si-O-Si) [49]. In hybrid materials, interestingly, the disappearance of most of the peaks is evident, while the downward or upward shift of different modes can be observed. For example, Si-O-Si breathing modes upshifted from 490 cm^−1^ to 505 cm^−1^, while C-H out-of-plane deformation peaks (600–850 cm^−1^) disappeared in the case of IGOPS and IPS nanomaterials. Impyridine@SiO_2_ maintains these peaks, probably because of the existence of two aromatic rings (pyridine plus imidazole), in contrast with IGOPS and IPS, which contain only imidazole. However, the bands that contributed to imidazole, i.e., v(C-Ν) (1310–1404 cm^−1^, deformation) and v(C-C) (1429–1781 cm^−1^, aromatic ring), maintained and upshifted to lower wavenumbers. Moreover, impyridine@SiO_2_ and IGOPS demonstrate a new peak at ~1190 cm^−1^, which can be attributed to the vibration mode of the ether group (v(C-O)) that they bear in their molecular structure (Figure S2a,c of the Supplementary Material). This mode, in combination with the upward shift of the intertetrahedral Si-O-Si vibration mode and the vibrations assigned to the aromatic rings, further confirms the successful synthesis of imidazole-based nanohybrids. 

### 3.2. FA Catalytic Dehydrogenation

#### 3.2.1. Optimization of Catalytic Procedure

To check if the sequence of chemicals’ addition affects the performance of catalysis, various experimental procedures were performed, as shown in Figure S6 of the Supplementary Material. The optimum was obtained when, to a propylene carbonate/FA mixture (5/2 *v*/*v*), the chemicals were added following the order: source of Fe^2+^, imidazole-based nanohybrid and PP_3_. Interestingly, when PP_3_ is not inserted last in the catalytic reaction, this highly reduces both gas production and the reaction rate (see Figure S6a,b). This effect could be attributed to the polydentate nature of PP_3_, which probably creates a saturated environment around Fe^2+^, preventing the approaching of other catalytic components [29]. Moreover, considering that PP_3_ is necessary to initiate gas evolution, it probably plays another role beyond its ligation, i.e., adjusting the solution’s potential for a catalytic reaction [51]. In addition, the molar ratio of [Fe^2+^/IGOPS material/PP_3_] was investigated (see Figure S6c); the optimum catalytic behavior is exhibited by the ratio [Fe^2+^/IGOPS material/PP_3_] = [7.5/15/7.5 μmol]. Homogeneous catalytic systems with imidazole or impyridine as nitrogen-based ligands are affected in the same way by the sequence of reagents’ addition and their molecular ratio; that is, the optimum is obtained by following the addition order of Fe^2+^, imidazole-based ligand and PP_3_ with the ratio [Fe^2+^/imidazole-based ligand/PP_3_] = [7.5/15/7.5 μmol]. Therefore, we maintained the above experimental conditions through the whole of our study. 

#### 3.2.2. Catalytic Results

Catalytic gas evolution, as monitored by GC-TCD, revealed that the produced gas consisted exclusively of H_2_ and CO_2_ with a constant ratio of [H_2_/CO_2_ = 1/1] during the catalytic reaction [14,17,18]. The present catalytic systems are highly selective, which is crucial for the applications of fuel cells, as no CO was detected. All the catalytic data presented herein were derived from the average of at least three experimentation sets with a standard error of 5%. Figure S7a,b of the Supplementary Material depict the gas volume produced by [Fe^2+^/IGOPS/PP_3_], [Fe^2+^/IPS/PP_3_] and [Fe^2+^/impyridine@SiO_2_/PP_3_] vs. the homogeneous imidazole and impyridine counterparts. Interestingly, a higher production rate was achieved when the imidazole was in the homogeneous phase, producing V(H_2_ + CO_2_) = 2380 mL within 40 min, which corresponds to a 100% yield. A 10% decrease in catalytic gas production was observed in the case of [Fe^2+^/IGOPS/PP_3_] (V(H_2_ + CO_2_) = 2142 mL within 40 min), while [Fe^2 +^/IPS/PP_3_] produces almost the same yield but at a lower rate (the reaction was completed in 75 min). In the case of impyridine@SiO_2_ (Figure S7b), the corresponding homogeneous impyridine had a performance of almost zero, producing only 20 mL of gas in total, in contrast with the homologous heterogeneous counterpart, which presents a satisfying catalytic activity of V(H_2_ + CO_2_) = 1750 mL within 55 min. We referred to this analogous behavior in our previous work [42], where we proved that the immobilization of PPh_3_ onto the SiO_2_ surface generates an active Fe^2+^/RPPh_2_@SiO_2_ heterogeneous catalytic system which produces up to 14 lt of H_2_, whereas the corresponding homogeneous Fe^2+^/RPPh_2_ was completely inactive; this was attributed to the considerable reduction in the activation energy step barrier which occurred after the ligand’s grafting onto SiO_2_ [42]. 

In the context of the study of the catalytic reaction and the effect that each component has on the potential of the solution, the solution potential, E_h_, (vs standard hydrogen electrode, SHE) was mapped, and the results are given in the following figure.

**Figure 5:**
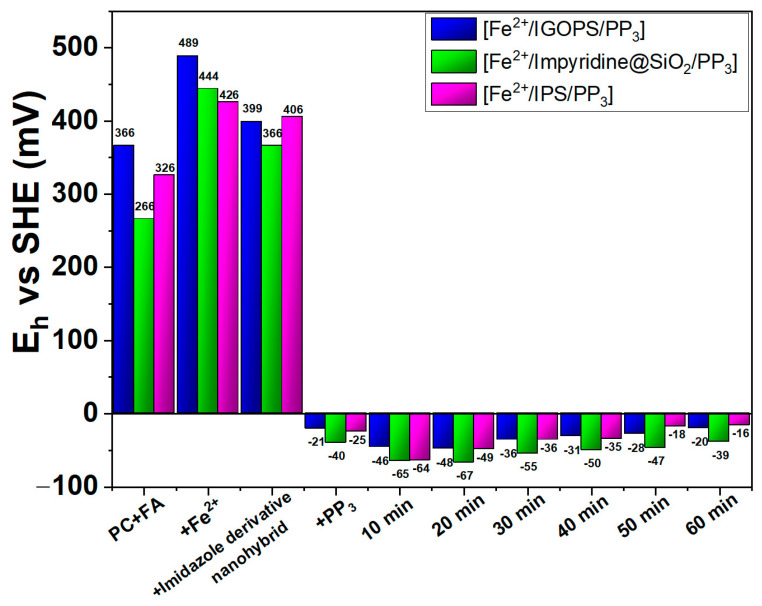
Mapping of redox potential of the solution (E_h_ vs. standard hydrogen electrode, SHE) for heterogeneous [Fe^2+^/IGOPS/PP_3_], [Fe^2+^/IPS/PP_3_], [Fe^2+^/impyridine@SiO_2_/PP_3_] catalytic systems, during the different stages of the reaction.

The data in Figure 5 show that, before the reaction began, the redox potential of the solution had positive values, indicating the highly oxidizing environment which is created by the solvent (propylene carbonate) and substrate (formic acid). The addition of Fe^2+^ changes E_h_ to more positive values, e.g., from +366 mV vs. SHE to +489 mV vs. SHE for the catalytic system [Fe^2+^/IGOPS/PP_3_]. A small decrease in E_h_ is observed when a IGOPS, IPS or impyridine@SiO_2_ nanohybrid containing reductive imidazole groups is added. Remarkably, the polydentate alkyl-phenyl-phosphine ligand (PP_3_) highly decreases E_h_, resulting in a reducing environment with slightly negative E_h_ values. This change is accompanied by gas generation, indicating the initiation of the catalytic reaction. After 10 min, E_h_ became more negative, with the [Fe^2+^/impyridine@SiO_2_/PP_3_] having a higher value (E_h_= −65 mV vs. SHE). As the reaction progressed, the E_h_ continued to present negative values, with those of the [Fe^2+^/impyridine@SiO_2_/PP_3_] system being the lowest. Homogeneous imidazole and impyridine counterparts present a similar trend, with the impyridine having more negative values (see Figure S8 of the Supplementary Material). From the above results, it seems that the poly-phosphine ligand (PP_3_) is necessary for reaction initiation, resulting in a negative Eh value. However, the generation of a reduced or a highly reduced environment does not ensure catalytic reactivity or/and performance—see, for example, the homogeneous [Fe^2+^/impyridine/PP_3_] system. From a mechanistic point of view, a slow and determining step for catalytic FA dehydrogenation is the β-H abstraction from the formate coordinated on the Fe-center [52], which is probably triggered by phosphine’s addition. As a result, CO_2_ is emitted and the Fe-H species is formed, which then is protonated, resulting in H_2_ production [53].

**Figure 6:**
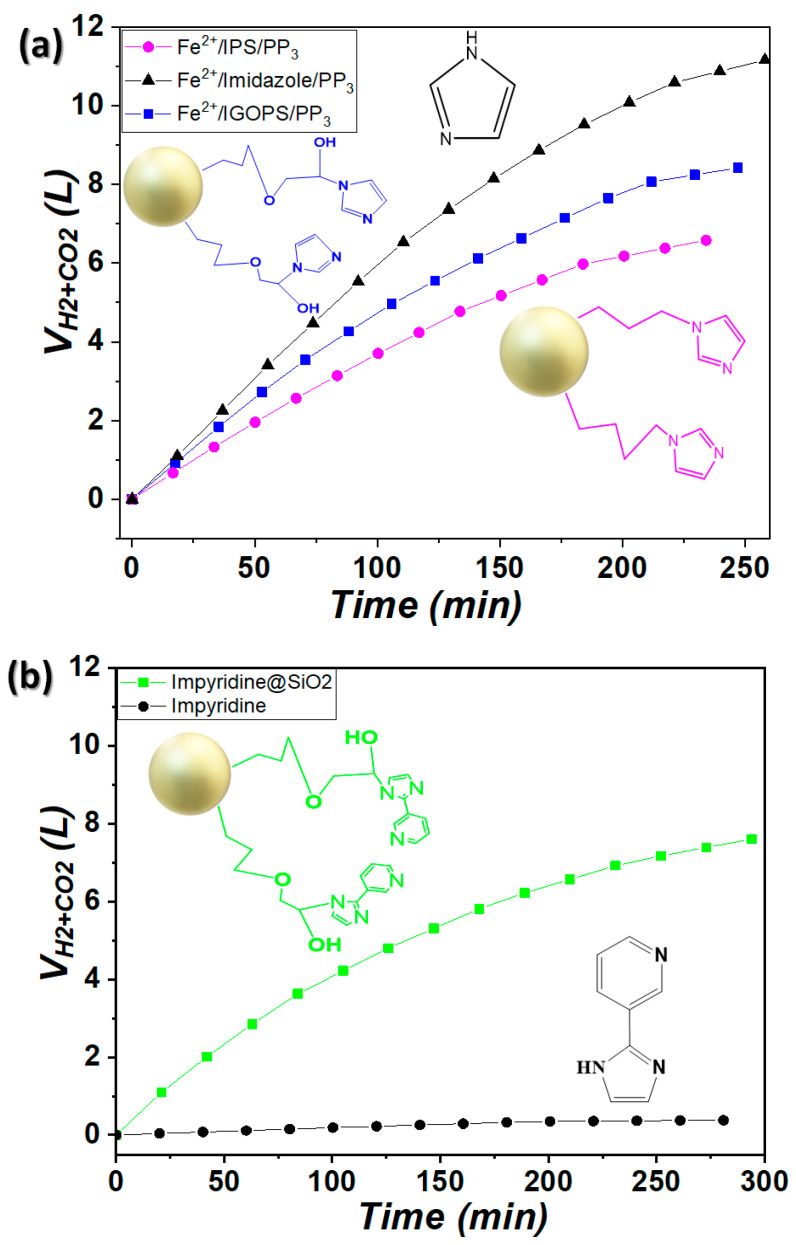
Gas evolution (VH_2_ + CO_2_), after the consecutive additions of FA catalyzed by [Fe^2+^/imidazole-based ligand/PP_3_]. (**a**) The nanohybrids IGOPS and IPS vs. homogeneous imidazole. (**b**) impyridine@SiO_2_ vs. homogeneous impyridine. Reaction conditions: [Fe^2+^/imidazole-based ligand/PP_3_] = [7.5/15/7.5] μmol, 7 mL of propylene carbonate/FA mixture (5/2 *v*/*v*), T = 80 °C (±1 °C), followed by consecutive additions of 1 mL of FA.

In order to investigate the performance of the catalytic systems upon the continuous feeding of FA, after the catalytic conversion of the initial 1 mL of FA, a new amount of FA (1 mL) was introduced to the catalytic reaction without any further addition of reagents. The catalytic gas production data in Figure 6a indicate that the [Fe^2+^/IGOPS/PP_3_] nanohybrid generated a total gas volume of V(H_2_ + CO_2_) = 8.42 L after the continuous addition of 9 mL FA, presenting total TONs = 22,953 and TOFs = 5571 h^−1^. Inferior performance, with a decrease of approximately 15%, was noted for the [Fe^2+^/IPS/PP_3_] (TONs = 17,938, TOFs = 4599 h^−1^, V(H_2_ + CO_2_) = 6.58 L). Comparatively, the generation rate of the homogeneous counterpart [Fe^2+^/imidazole/PP_3_] showed higher TONs and TOFs (see Table 2). On the other hand, the homogeneous impyridine had at almost zero catalytic efficiency in comparison with heterogeneous impyridine@SiO_2_, which had satisfactory activity, with TOFs = 4228 h^−1^ (TONs= 20,718, V(H_2_ + CO_2_) = 7.6 L).

Indeed, in all catalytic systems, the gas evolution was stopped after a satisfactory amount of FA was added (e.g., for [Fe^2+^/IGOPS/PP_3_], after the addition of 9 mL of FA). This could be due to the accumulation of H_2_O, which is present at a concentration of 2.5% [*v*:*v*] in the FA stock obtained from the supplier, as we examined in our previous work [42]. To verify this hypothesis here, in the catalytic system [Fe^2+^/IGOPS/PP_3_] upon normal operation conditions, small quantities of H_2_O were added. The inhibiting role of H_2_O was confirmed, since the gas production rate diminished after the addition of 200 μL of H_2_O and ceased when a total amount of 400 μL was added (Figure S9, Supplementary Material).

In homogeneous catalytic systems (imidazole or impyridine), the suppressive impact of H_2_O is not easily overcome. Nevertheless, as we demonstrate hereafter, the current heterogeneous systems provide a low-cost solution for removing this inhibiting effect: after H_2_ production ceased, e.g., after continuous H_2_ production from 9 mL of FA by [Fe^2+^/IGOPS/PP_3_], 7 mL of FA by [Fe^2+^/IPS/PP_3_], and 8 mL of FA by [Fe^2+^/impyridine@SiO_2_/PP_3_], the suspension was centrifuged and rinsed, and the resulting catalyst was reused, resulting in continuous H_2_ production from new quantities of added FA (Figure 6). This demonstrates that the decrease in catalytic performance after the dehydrogenation of 8 mL of FA approximately was not due to irreversible damage of the catalyst, as it can be resolved by means of a straightforward washing and drying procedure. 

**Figure 7:**
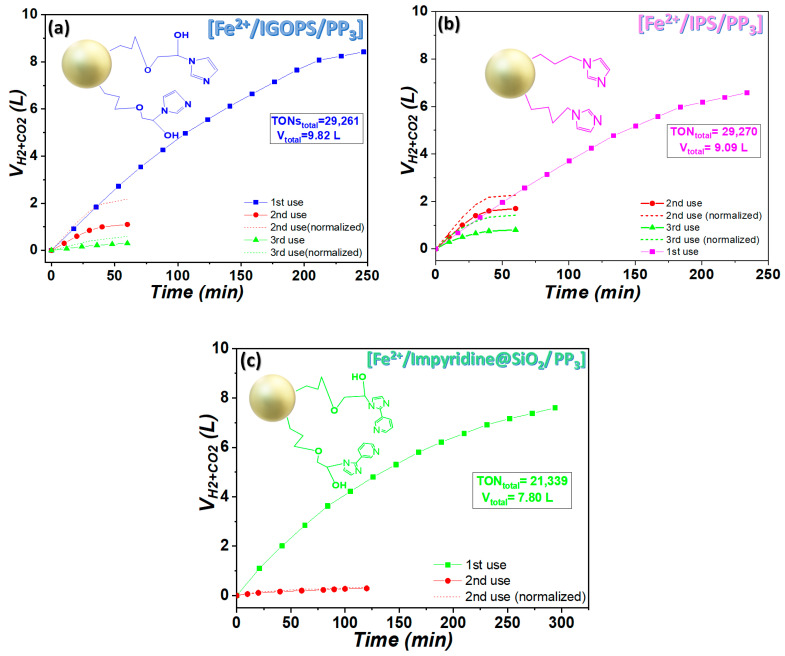
Recyclability of [Fe/imidazole derivatives/PP_3_] heterogeneous systems: (**a**) IGOPS, (**b**) IPS, (**c**) impyridine@SiO_2_. Reaction conditions: [Fe^2^/imidazole-based ligand/PP_3_] = [7.5/15/7.5] μmol, 7 mL propylene carbonate/FA mixture (5/2 *v*/*v*), T = 80 °C (±1 °C) followed by consecutive additions of 1 mL of FA.

Within this context, we recovered and reused the present heterogeneous systems. Thus, when catalytic gas evolution stopped, the solid catalyst was collected via centrifugation, washed with methanol and employed for a second use under the same catalytic conditions, with no further Fe addition. In the case of the IGOPS nanohybrid (see Figure 7a), 41.6 mg was recovered and applied for a second use, producing 1.1 L of gases within 1 h, with TONs = 5597 and TOFs = 5597 h^−1^, and an average production rate 30 mL/min (Table 3). Subsequently, when the catalysis stopped again, 21 mg of the solid catalyst could be recovered, washed, and applied for a third reuse, providing TONs = 3228 within 1 h (Table 3). Overall, the [Fe^2+^/IGOPS/PP_3_] system was reused three times with no further [Fe^2+^/IGOPS] addition, providing 9.82 L of gases and 31,780 TONs, taking into account the fact that the best performance is achieved within the first use (Table 3). On the contrary, [Fe^2+^/IPS/PP_3_] presents an inferior performance during the first use, but it seems that it maintains its efficiency after the second and third use by recycling 62.7 and 35.3 mg, respectively, and producing, in total, 9.1 L of gases and 29,260 TONs (Figure 7b, Table 3). Nanohybrid impyridine@SiO_2_ was practically non-reusable. 

Based on the data in Table 3, the catalytic performance of reused [Fe^2+^/IGOPS/PP_3_] gradually reduced. To check if the observed loss of activity is due to the loss of catalyst mass, the volume of gas produced upon recycling runs, i.e., the second and third use, was normalized based on the nanohybrid material mass used for the first run (see Figure 7a dotted lines). This data analysis demonstrates that other reasons beyond mass catalyst loss are responsible for catalyst deactivation. 

In order to investigate the drop in catalytic efficiency in the case of [Fe^2+^/IGOPS/PP_3_] and [Fe^2+^/impyridine@SiO_2_/PP_3_], two independent protocols were established;

(i) Monitoring leaching of Fe^2+^ species through the solution after the end of the reaction using UV/Vis spectroscopy. According to [54] and our previous study [18], the UV/Vis spectra of Fe^2+^/PP_3_ complexes exhibit a prominent peak at 510 nm (as depicted in Figure S10 of the Supplementary Material), which is attributed to the occurrence of MLCT transitions [55]. When removing the solid catalyst after the end of the reaction by means of filtration, the characteristic band at 510 nm did not appear, proving that the decrease in catalytic efficiency is not attributed to the leaching of Fe^2+^ atoms.
(ii) Using FT-IR and Raman spectroscopy, as long as the reaction was completed and the catalytic materials were recovered, it is revealed that the C-O bond (1320 cm^−1^, FT-IR and 1190 cm^−1^, Raman) of IGOPS and impyridine@SiO_2_ vanished (Figures S11a–c and S12a–c of the Supplementary Material). IGOPS and impyridine@SiO_2_ bear a glycidyl group with a C-O-C bond due to the grafting method applied, while IPS only bears a propyl group, respectively (see Figure S2 of the Supplementary Material). It seems that the C-O-C bond is less stable in reducing conditions, and as a result, a release of [Fe^2+^-L, where L = imidazole derivative ligand] occurred, demonstrating the lower catalytic activity of IGOPS during the second and third use and the non-reusability of the impyridine@SiO_2_ system.

#### 3.2.3. Comparison of [Fe-Imidazole@SiO_2_] Nanohybrids with Other Immobilized Catalysts

The first heterogeneous systems used for FA dehydrogenation were metal particles—not complexes—[56] primarily operating at high temperatures (T > 200 °C) and pressures, with FA in gas form. Since 2008, liquid phase reactions that can proceed at near-ambient temperatures have been presented by the scientific community [57]. Utilizing the benefits of homogeneous catalytic metal complexes, an alternative strategy is the grafting of the metal complex onto a solid matrix. The first attempt to do this was made by the research group of Laurenczy in 2009 [39], immobilizing, by means of various techniques such as ion exchange and adsorption, an homogeneous Ru[meta-trisulfonatedtriphenylphospine] complex on different supports, including polymers and zeolites. In some instances, satisfactory catalytic activity was obtained, with a higher TOF of approximately 427 h^−1^ observed for the zeolite PB Na-BEA (Table 4). Leaching of the catalytically active complex from the surface was a significant disadvantage, as it caused the progressive deactivation of the catalyst. In order to overcome these limitations, the same research group, in a more recent work, immobilized a Ru(II)-phosphine catalyst onto mesoporous silica supports. The heterogenous catalytic complex MCM41-Si-(CH_2_)_2_PPh_2_/Ru-mTPPTS achieved a TOF = 2780 h^−1^ within 150 min [58]. Another example of an immobilized catalyst consisting of a Ru metal center with sulfur ligands covalently bonded to a SiO_2_ support was the Ru-S-SiO_2_ compound with a moderate activity of TOF = 344 h^−1^ [59]. Ir immobilized complexes on SiO_2_ matrixes were shown to be the most promising, with TOFs > 10,000 h^−1^ [40,41], despite the high cost [8]. The impact of the central metal cation (Rh and Ir) was investigated by Yoon et al. [60] using half-sandwich Rh(III) or Ir(III) catalysts immobilized on bipyridine-based covalent triazine frameworks with tunable dimensions (bpy-CTFs). They found that Ir_4.7_@bpy-CTF400 and Rh_1.7_@bpy-CTF400 heterogenous catalysts presented the highest H_2_ yields, with initial TOFs = 2860 h^−1^ and 1760 h^−1^, respectively. To the best of our knowledge, our research group was the first time to present a cheap non-noble metal [Fe-phosphine@SiO_2_] catalyst with satisfactory performance (TONs = 8041 and TOFs = 4308 h^−1^ for Fe/polyRPhphos@SiO_2_) [42]. Herein, we presented heterogeneous [Fe/imidazole derivatives@SiO_2_/PP_3_] catalytic systems, with higher activity and stability. [Fe/IGOPS] presented TONs = 22,953 and TOFs = 5571 h^−1^, within 247 min. Comparing the data in Table 4, it is shown that the Fe catalytic complexes of the present work surpass the activity of the precious Ru immobilized catalysts by almost two times (TOFs_[Fe/IGOPS]_ = 5571 h^−1^ vs. TOFs_MCM41-Si-(CH2)2PPh2/Ru-mTPPTS_ = 2780 h^−1^ in [58]). The high-cost Ir complexes of [Cp*Ir(pyridylimidazoline)(H_2_O)]@SiO_2_ [40] and Ir_PicaSi_SiO_2_ [41] seem to be more effective, with TOFs >11,000 h^−1^, but have lower stability (TONs_[Fe/IGOPS]_ = 22,953 vs. TONs_Ir_PicaSi_SiO2_= 17,600 [41]). Overall, it seems that the present low-cost imidazole-based nanohybrids IGOPS and IPS linked with the non-noble Fe^2+^ metal may constitute heterogeneous catalytic systems with excellent stability and performance for FA dehydrogenation.

## 4. Conclusions

Three highly efficient heterogeneous catalytic systems ([Fe^2+^/IGOPS/PP_3_], [Fe^2+^/IPS/PP_3_] and [Fe^2+^/impyridine@SiO_2_/PP_3_]) have been developed for H_2_ generation from FA by covalently grafting an Fe^2+^-imidazole derivative onto a mesoporous nano-SiO_2_ matrix. BET and HRTEM revealed that the immobilization of the imidazole derivative onto the SiO_2_ has an important impact to the SSA, average pore volume, and particle size distribution. Their catalytic activity (TONs, TOFs), stability, and reusability in the context of FA dehydrogenation were evaluated. The homologous homogeneous counterparts are also evaluated for the purpose of comparison. Mapping of the redox potential of solution E_h_ demonstrated the essential role of poly-phosphine PP_3_ in FA dehydrogenation. On the basis of performance and stability, [Fe^2+^/IGOPS/PP_3_], exhibited superior behavior in comparison to the other heterogeneous catalysts, producing 9.82 L of gases (VH_2_ + CO_2_) with TONs= 31,778, despite a decrease in efficiency after the first use. In contrast, [Fe^2+^/IPS/PP_3_] demonstrated the maximum level of recyclability, retaining its performance after three consecutive uses, providing 9.09 L of gases (VH_2_ + CO_2_) with TONs= 29,252. With V_H2+CO2_ = 7.8 L, the [Fe^2+^/impyridine@SiO_2_/PP_3_] had a diminished activity and was not reusable. Interestingly, the homogeneous equivalent [Fe^2+^/impyridine/PP_3_] was completely inactive. The drop in catalytic efficiency observed upon continuous feeding of FA was investigated and attributed to the accumulation of H_2_O, derived from the FA stock which contains 2.5% water. In the case of [Fe^2+^-imidazole@SiO_2_] nanohybrids, the catalysis’ inhibition by H_2_O is reversible, and it can be overcome by the simple washing and drying of nanohybrids. Raman, FT/IR, and UV/Vis spectroscopy demonstrate that the lower recyclability of [Fe^2+^/IGOPS/PP_3_] and [Fe^2+^/impyridine@SiO_2_/PP_3_] nanohybrids is attributable to the cleavage of their C-O-C bonds in the reducing conditions during catalysis. Overall, it was demonstrated that the low-cost imidazole-based nanohybrids such as IGOPS and IPS associated with the non-noble Fe^2+^ metal are able to form [Fe^2+^/IGOPS/PP_3_] and [Fe^2+^/IPS/PP_3_] heterogeneous catalytic systems, respectively, with high stability and performance for FA dehydrogenation. It seems that nanohybrid catalysts can offer an efficient, convenient and low-cost alternative in H_2_ production based on the catalytic dehydrogenation of C1 molecules. 

## Figures and Tables

**Table 1:** Physicochemical characteristics of imidazole@nanosilica derivatives.

Nanomaterial	Organic Loading (mmol/g_nanosilica_)	d_TEM_ (nm)	SSA (m^2^/g)	Average Pore Volume (cc/g)
SiO_2_	-	25	398	0.71
IGOPS	0.82	26	304	0.41
IPS	0.45	28	303	0.45
Impyridine@SiO_2_	0.24	35	310	0.59

**Table 2:** Catalytic results of [Fe^2+^/imidazole-based ligand/PP_3_] systems for FA dehydrogenation, using a continuous operation mode.

Catalytic System	V_H2+CO2_(L)	TONs	TOFs (h^−1^)	V_FA_ (mL)	Rate (mL/min)
**[Fe^2+^/IGOPS/PP_3_]**	8.42	22,953	5571	9	52
**[Fe^2+^/IPS/PP_3_]**	6.58	17,938	4599	7	40
**[Fe^2+^/Impyridine@SiO_2_/PP_3_]**	7.60	20,718	4228	8	45
**[Fe^2+^/Imidazole/PP_3_]**	9.00	24,535	5705	9	55
**[Fe^2+^/Impyridine/PP_3_]**	0.38	138	29	4	2

Reaction conditions: [Fe^2 +^/imidazole-based ligand/PP_3_] = [7.5/15/7.5] μmol, 7 mL of propylene carbonate/FA mixture (5/2 *v*/*v*), T = 80 °C (±1 °C). After the production of 1200 mL of gasses, 1 mL (26 mmol) of FA was added.

**Table 3:** Catalytic results of [Fe/imidazole derivatives@SiO_2_/PP_3_] systems after consecutive uses.

[Fe/IGOPS]	Mass (mg)	Reaction Time (min)	V_H2+CO2_ (L)	Rate (mL/min)	TONs	TOFs	V_H2+CO2_ (L)_normalized_
1st use	81.9	247	8.42	52	22,953	5571	8.42
2nd use	41.6	120	1.10	30	5597	2799	2.17
3rd use	20.0	60	0.30	6.7	3228	3228	0.60
TOTAL	-	-	9.82	-	31,778	-	11.19
**[Fe/IPS]**	**Mass (mg)**	**Time (min)**	**V_H2+CO2_ (L)**	**Rate (mL/min)**	**TONs**	**TOFs**	**V_H2+CO2_ (L)_normalized_**
1st use	83.5	234	6.58	40	17,938	4599	6.58
2nd use	62.7	60	1.70	50	6163	6163	2.26
3rd use	35.3	60	0.80	30	5151	5151	1.42
TOTAL	-	-	9.09	-	29,252	-	10.26
**[Fe/Impyridine@SiO_2_]**	**Mass (mg)**	**Time (min)**	**V_H2+CO2_ (L)**	**Rate (mL/min)**	**TONs**	**TOFs**	**V_H2+CO2_ (L)_normalized_**
1st use	86.2	294	7.60	45	20,794	4228	7.6
2nd use	66.0	60	0.20	4	545	545	0.25
TOTAL	-	-	7.80	-	21,339	-	7.85

**Table 4:** Comparison of [Fe/imidazole derivatives@SiO_2_/PP_3_] with immobilized catalytic complexes from the pertinent literature.

Catalytic Complex	Temp. (°C)	Operation Time (min)	TONs	TOFs (h^−1^)	Substrate/Solvent/Additive	Ref.
[Ru–TPPTS]@PB Na-BEA	90	25	-	427	FA/H_2_O/HCOONa	[39]
MCM41-Si-(CH_2_)_2_PPh_2_/Ru-mTPPTS	120	150	-	2780	FA/H_2_O/HCOONa	[58]
Ru-S-SiO_2_	85	6000	-	344	FA/H_2_O/HCOONa	[59]
[Cp*Ir(pyridylimidazoline)(H_2_O)]@SiO_2_	60	480	-	11,830	FA/H_2_O	[40]
Ir_PicaSi_SiO_2_	40	420	17,600	11,200	FA/H_2_O/HCOOK	[41]
Ir_4.7_@bpy-CTF400	80	30	-	2860	FA/H_2_O	[60]
Rh_1.7_@bpy-CTF400	80	48	-	1760	FA/H_2_O	[60]
Fe/RP * Ph *_ 2 _ @SiO_2_	80	140	7796	3341	FA/PC	[42]
Fe/polyRPhphos@SiO_2_	80	112	8041	4308	FA/PC	[42]
[Fe/IGOPS]	80	247	22,953	5571	FA/PC	This work
[Fe/IPS]	80	234	17,938	4599	FA/PC	This work
[Fe/Impyridine@SiO_2_]	80	294	20,794	4228	FA/PC	This work

## Supplementary Materials

The following supporting information can be downloaded at: https://www.mdpi.com/article/10.3390/nano13101670/s1, Figure S1: Schematic illustration of the synthesis of a impyridine@SiO_2_ nanohybrid, Figure S2: Molecular structures and photos of functionalized nano-SiO_2_ hybrid materials. (a) IGOPS. (b) IPS. (c) Impyridine@SiO_2_, Figure S3: N_2_ adsorption–desorption isotherms of (a) SiO_2_, (b) IGOPS, (c) IPS, and (d) impyridine@SiO_2_ hybrid materials, Figure S4: Pore size distribution plot using the BJH method. (a) SiO_2_. (b) IGOPS. (c) IPS. (d) Impyridine@SiO_2_, Figure S5: Particle size distribution of nano-SiO2 hybrids. (a) SiO_2_. (b) IGOPS. (c) IPS. (d) Impyridine@SiO_2,_ Figure S6: Optimization of catalytic conditions. (a) Total gas volume (H_2_ + CO_2_) and (b) TONs and TOFs obtained with different orders of reagents’ addition. (c) Total gas volume (H_2_ + CO_2_) as a function of the molar ratio of [Fe^2+^/IGOPS material/PP_3_]. Dotted line: maximum theoretical production of gasses (H_2_ + CO_2_), adding 52 mmol (2 mL) of FA, Figure S7: Gas volume (H_2_ + CO_2_) evolution, adding 2 mL of FA. (a) IGOPS and IPS vs. homogeneous imidazole. (b) Impyridine@SiO_2_ vs. homogeneous impyridine. Dotted line: maximum theoretical production of gasses (H_2_ + CO_2_), adding 52 mmol (2 mL) of FA, Figure S8: Solution redox potential, E_h_ (mV vs. SHE) values for the homogeneous catalytic systems [Fe^2+^/imidazole/PP_3_] and [Fe^2+^/impyridine/PP_3_], Figure S9: The impact of water on the catalytic performance of [Fe^2+^/IGOPS/PP_3_]. Total volume of H_2_O = 0.4 mL, Figure S10: Leaching test of Fe^2+^ after the end of the reaction for the catalytic system [Fe^2+^/IGOPS/PP_3_], Figure S11: Raman spectra of (a) IGOPS, (b) IPS, and (c) impyridine@SiO_2_ nanohybrids after reuse experiments, Figure S12: FT/IR of (a) IGOPS, (b) IPS, and (c) impyridine@SiO_2_ nanohybrids after reuse experiments.
