# Supplementary material for: Efficient [Fe-Imidazole@SiO2] Nanohybrids for Catalytic H2 Production from Formic Acid
Source: Nanomaterials (Basel). 2023 May 18;13(10):1670. doi: 10.3390/nano13101670 (PMC10222228; doi:10.3390/nano13101670)
Supplement: Supplementary file 1 [file nanomaterials-13-01670-s001.zip › nanomaterials-2398148-SI.pdf]

Supporting Information

Efficient [Fe-Imidazole@SiO_2] nanohybrids for catalytic H_2 production from formic acid

Christos Gatziouras, Maria Solakidou and Maria Louloudi *

Laboratory of Biomimetic Catalysis & Hybrid Materials, Department of Chemistry, University of Ioannina, 45110 Ioannina, Greece

* Correspondence: mlouloud@uoi.gr

Synthesis of Impyridine@SiO_2 nanohybrid

For the preparation of Impyridine@SiO_2, 0.663 ml of 3-(glycidyloxypropyl)-trimethoxysilane (3 mmol) was added to a solution of 2-(1H-Imidazol-2-yl)pyridine (3 mmol, 0.204 g) in methanol (50 ml). The resulting mixture was heated at 80 °C, under reflux, for 24 h to produce the silane precursor based on 2-(1H-Imidazol-2-yl)pyridine. Then 1.5 g of nano-SiO_2 and 5 ml of EtOH were added. The solution was stirred at 80 °C, under reflux, for an additional 24 h, resulting in the imidazole-modified material (hereafter referred to as Impyridine@SiO_2), which was filtered, rinsed with EtOH, and dried for 12 hours.

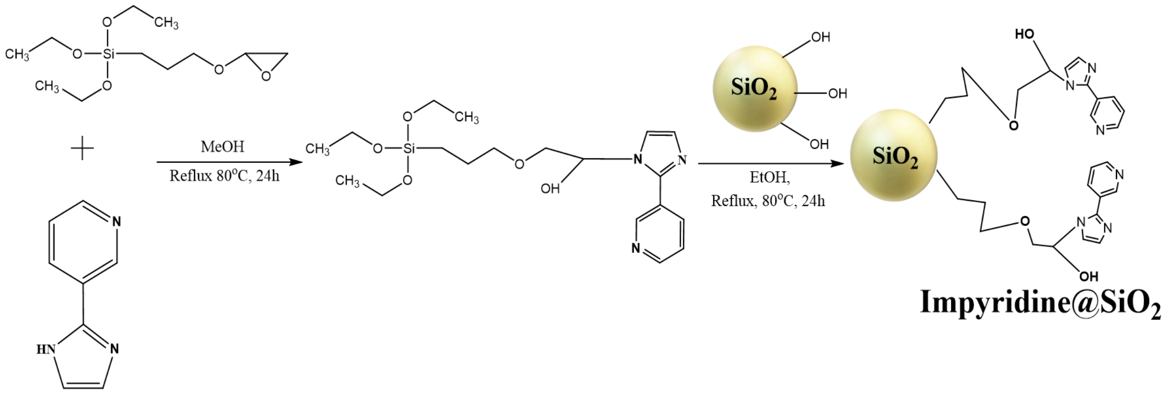

Figure S1. Schematic illustration of the synthesis of Impyridine@SiO_2 nanohybrid.

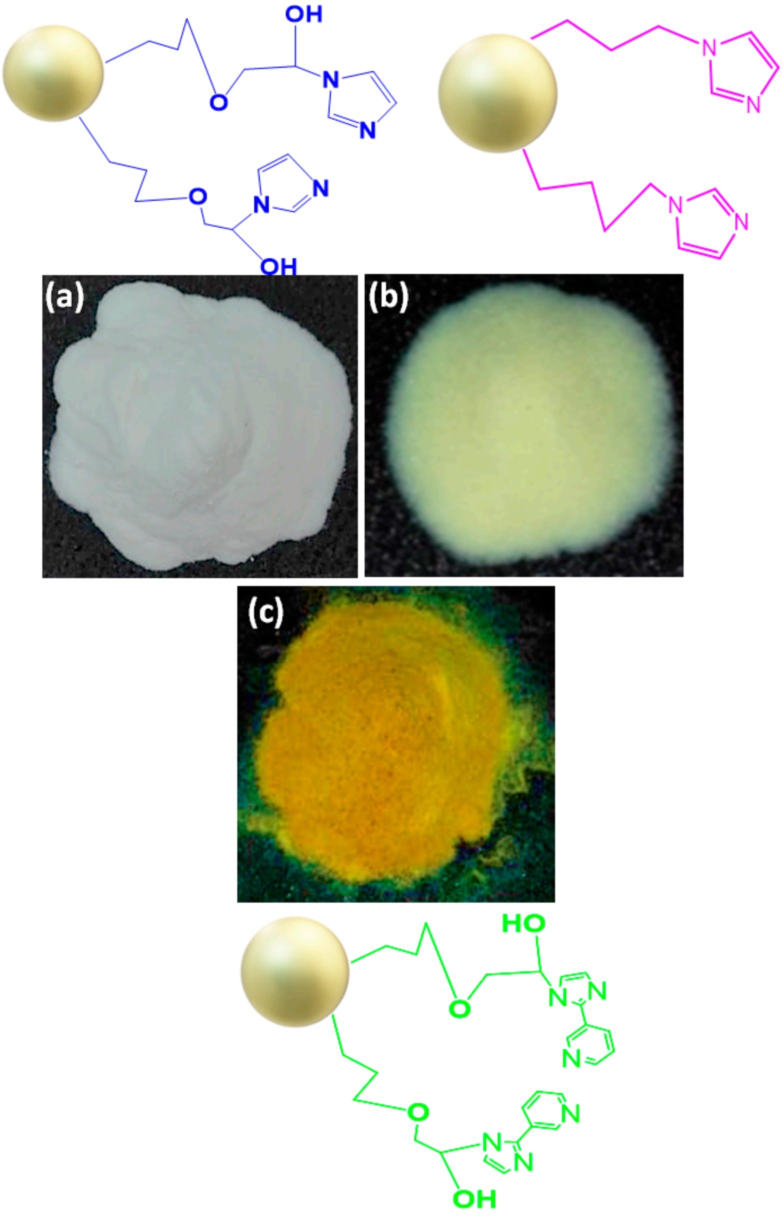

Figure S2. Molecular structures and photos of functionalized nano-SiO_2 hybrid materials (a) IGOPS (b) IPS (c) Impyridine@SiO_2

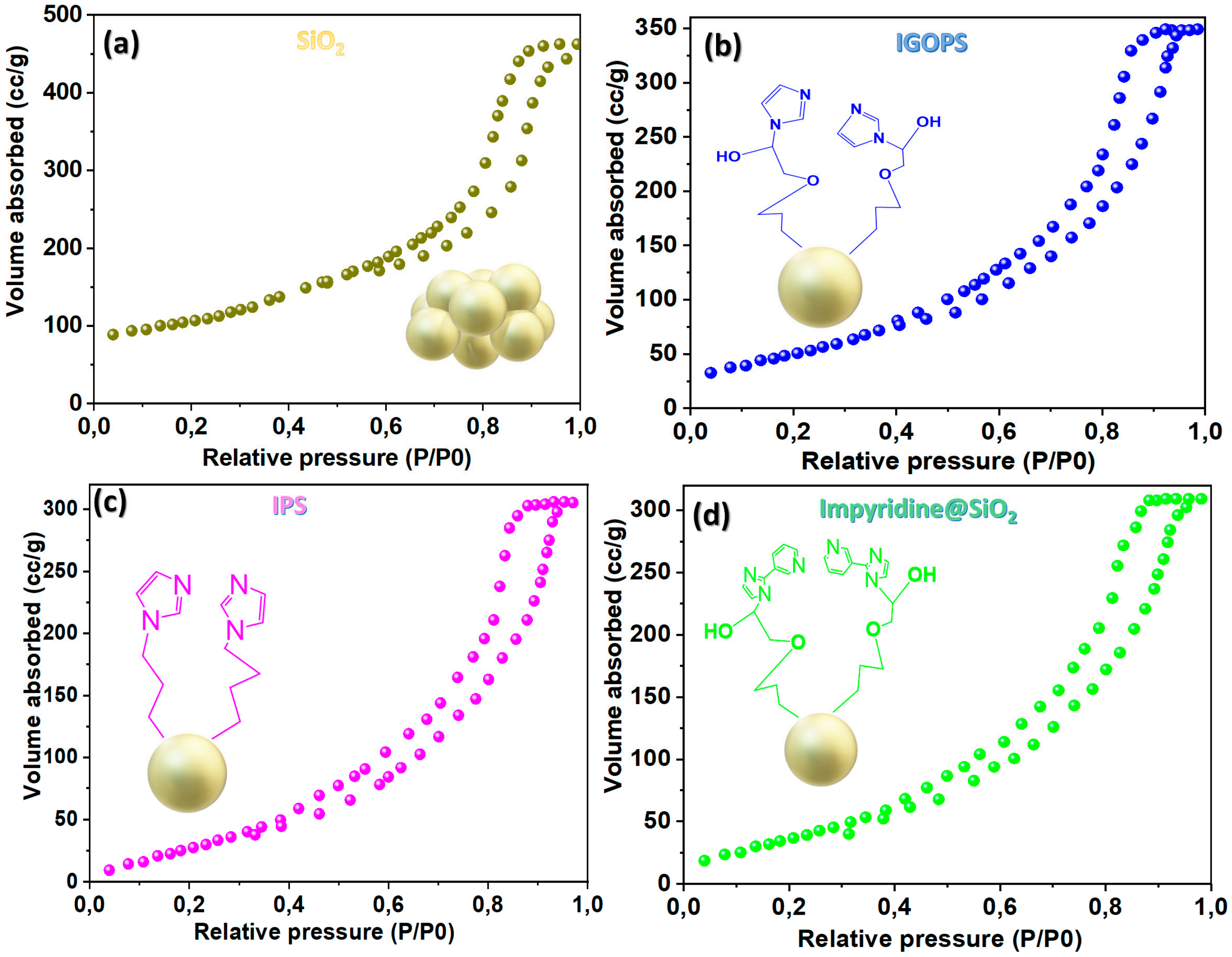

Figure S3. N_2 adsorption-desorption isotherms of (a) SiO_2 (b) IGOPS (c) IPS (d) Impyridine@SiO_2 hybrid materials

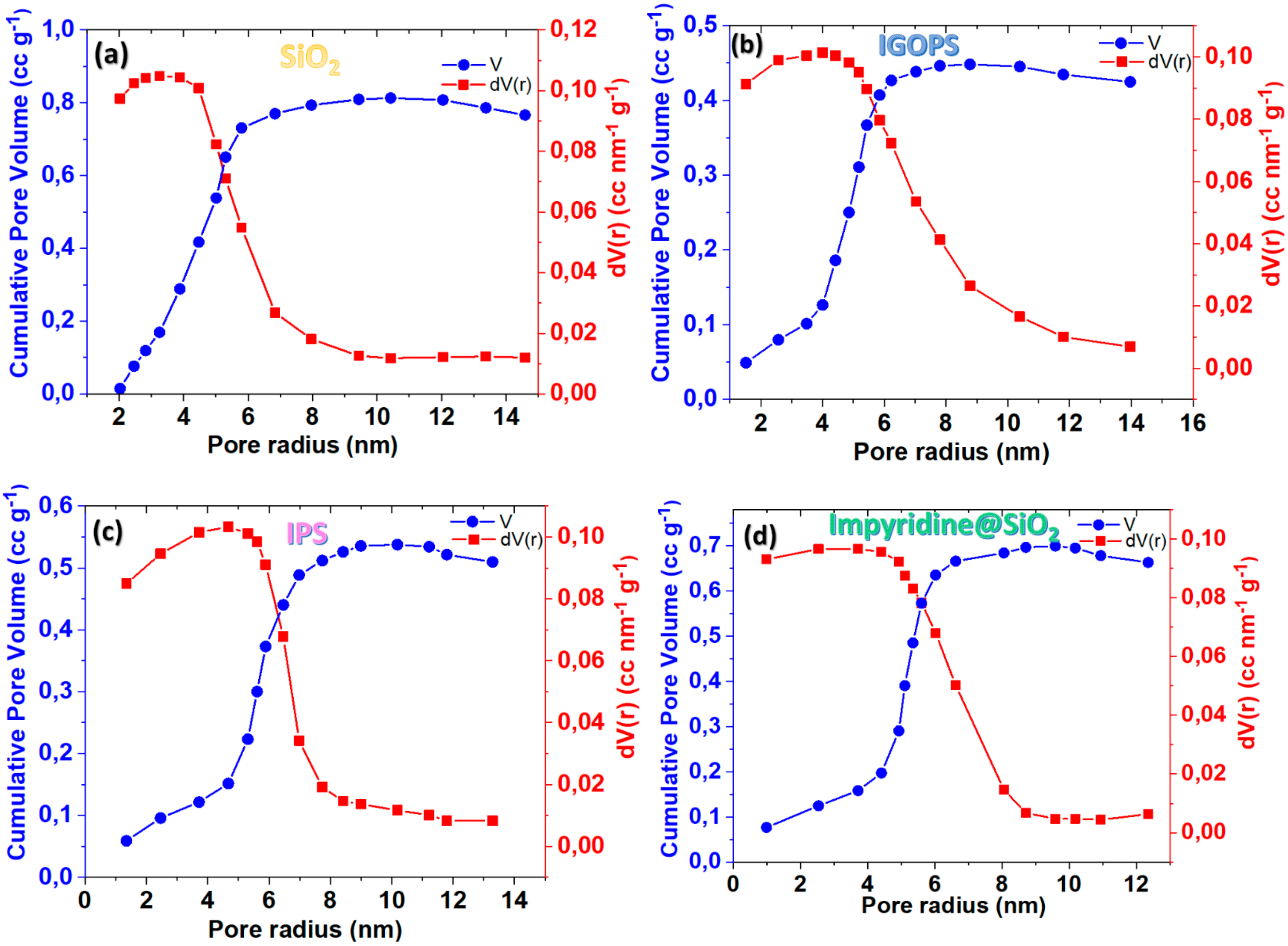

Figure S4. Pore size distribution plot using the BJH method. (a) SiO_2 (b) IGOPS (c) IPS (d) Impyridine@SiO_2

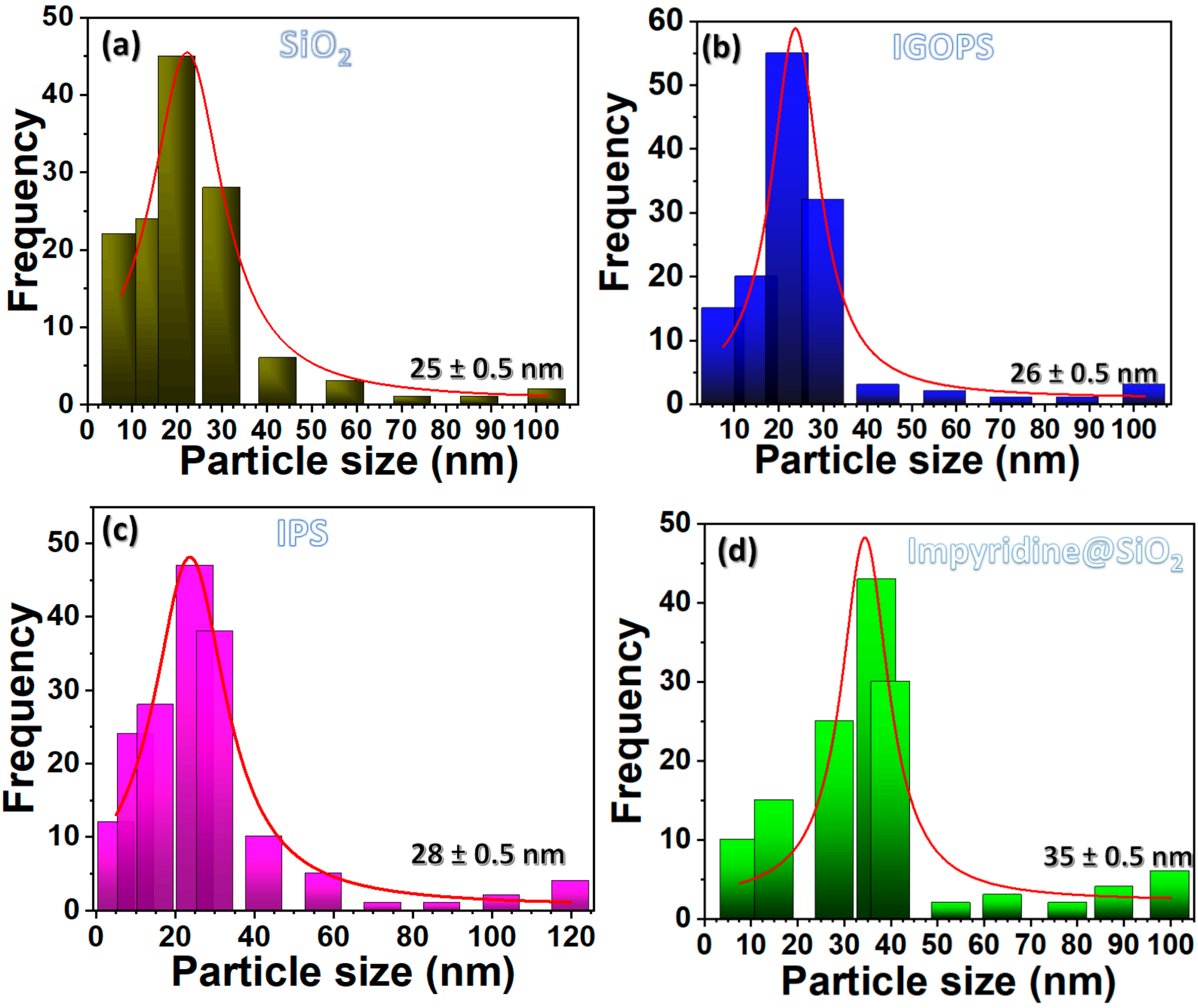

Figure S5. Particle size distribution of nano-SiO_2 hybrids; (a) SiO_2 (b) IGOPS (c) IPS (d) Impyridine@SiO_2

Equations for the calculation of TONs and TOFs

The Turnover Numbers (TON) and Turnover Frequencies (TOF)s were calculated using the following equations:

$$TON = \frac{V(H_2+CO_2)}{\frac{V_{mH_{2,25°C}}+V_{mCO_{2,25°C}}}{nRu}} \quad \textbf{(S1)}$$

$$TOF = \frac{TON}{t} \quad \textbf{(S2)}$$

where $V_{mH_{2,25°C}}+V_{mCO_{2,25°C}}$ and $V_{mCO_{2,25°C}}$ are the molar volume of H_2 and CO_2 at T=298 K, and P=1 atm, where $V_{mH_{2,25°C}}$ =24.49 L/mol and $V_{mCO_{2,25°C}}$ =24.42 L/mol.

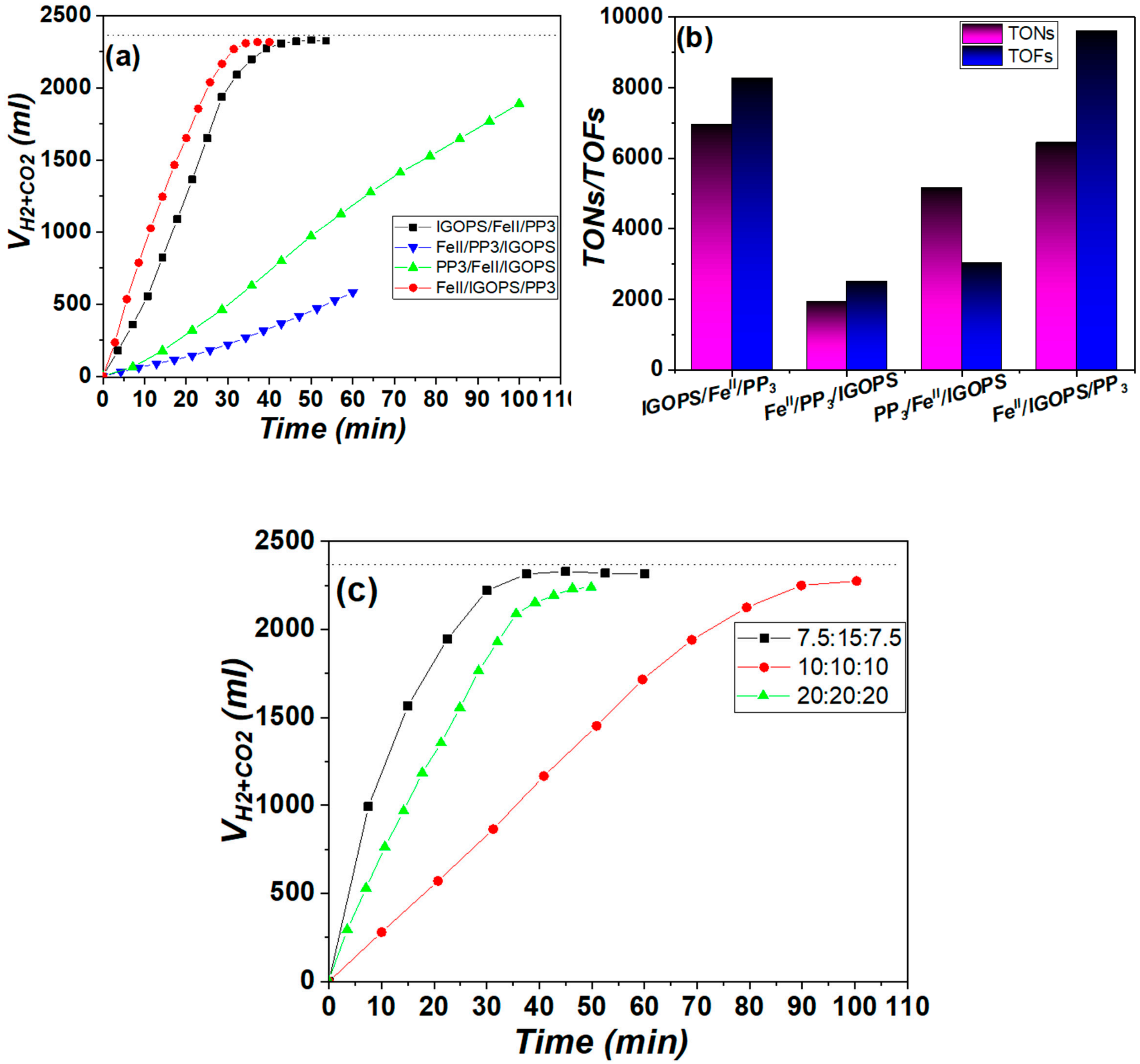

Figure S6. Optimization of catalytic conditions (a) Total gas volume (H_2+CO_2) and (b) TONs, TOFs obtained with different order of reagents' addition (c) Total gas volume (H_2+CO_2) as a function of the molar ratio of [Fe^{2+}/IGOPS material/PP_3]. Dotted line; maximum theoretical production of gasses (H_2+CO_2), adding 52 mmol (2 ml) of FA.

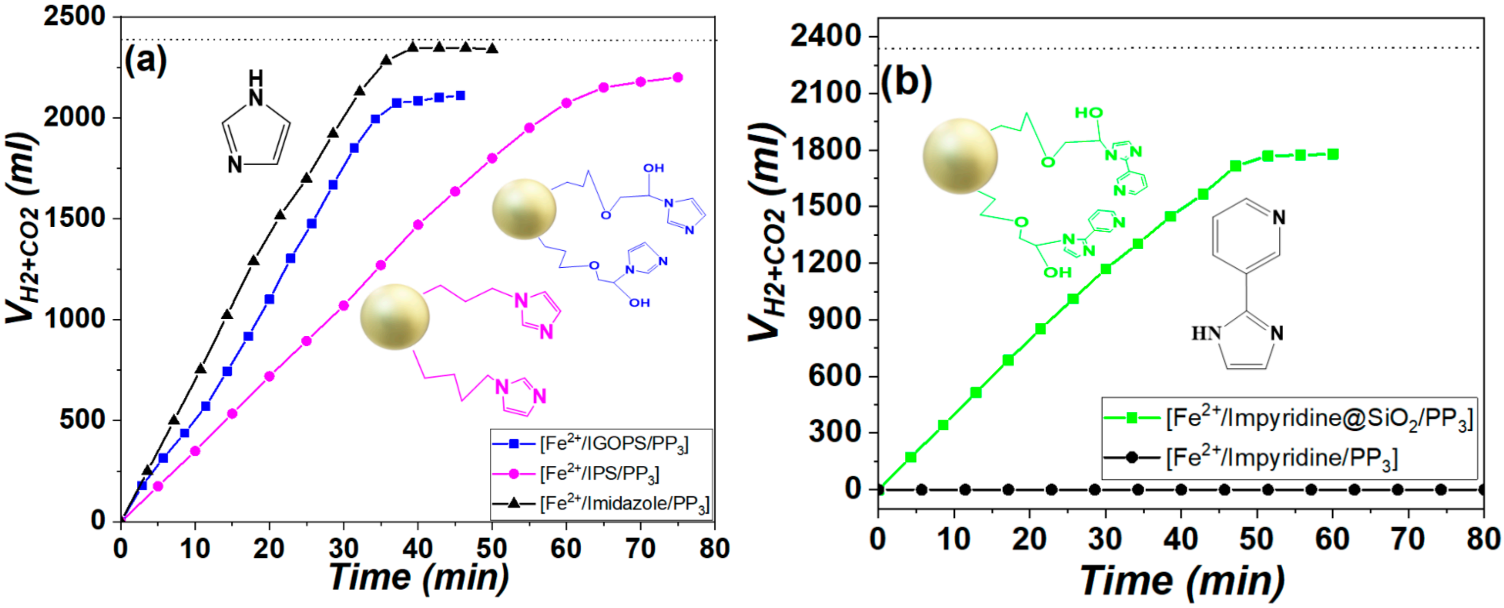

Figure S7. Gas volume (H_2+CO_2) evolution, adding 2 ml of FA (a) IGOPS, IPS vs homogeneous Imidazole (b) Impyridine@SiO_2 vs homogeneous Impyridine. Dotted line; maximum theoretical production of gasses (H_2+CO_2), adding 52 mmol (2 ml) of FA.

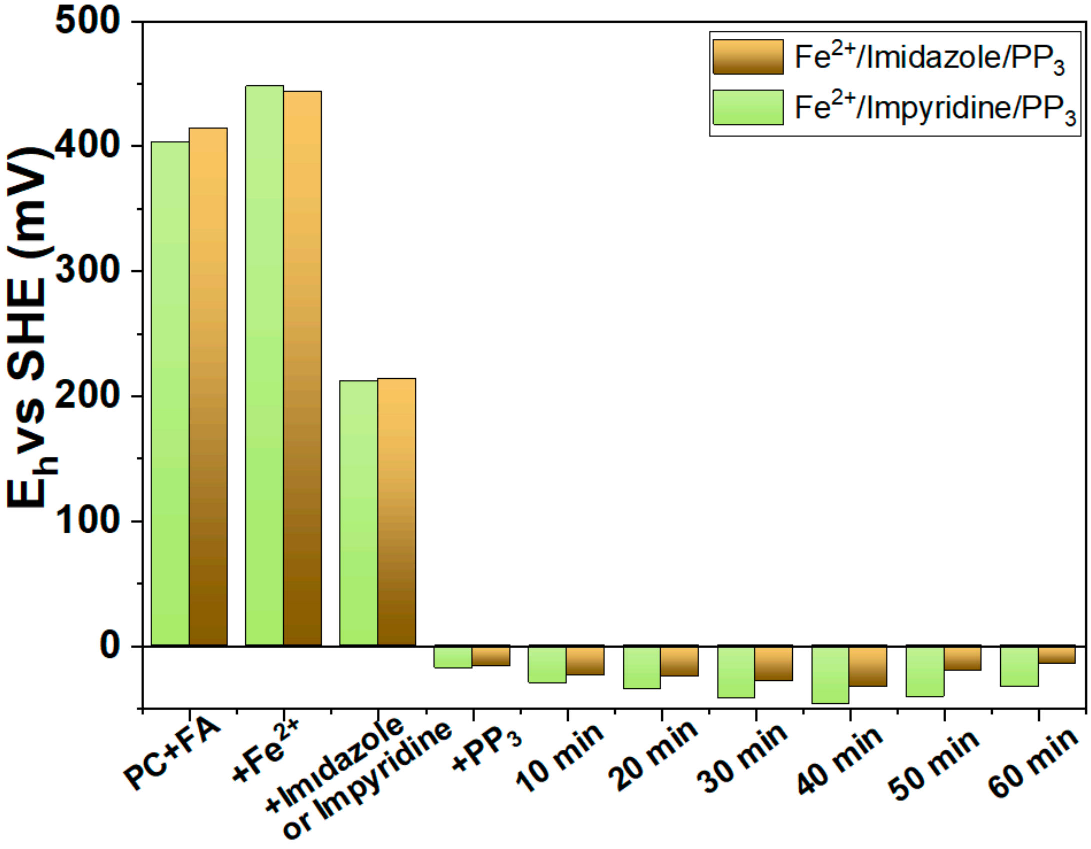

Figure S8. Solution redox potential, E_h (mV vs SHE) values for the homogeneous catalytic systems [Fe^{2+}/Imidazole/PP_3] and [Fe^{2+}/Impyridine/PP_3]

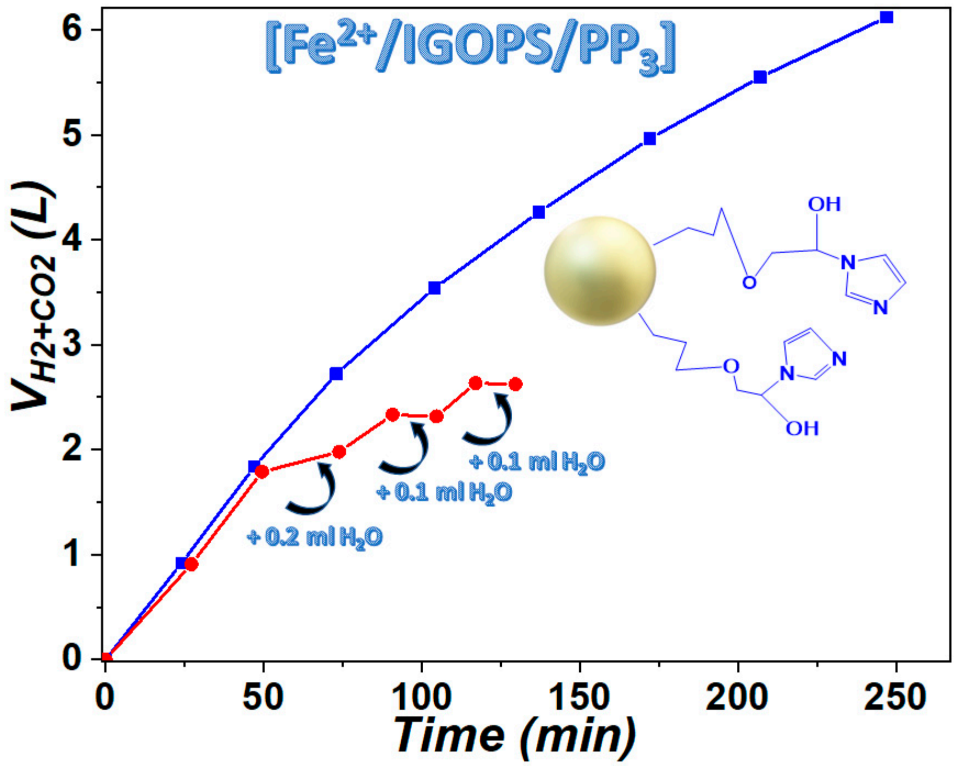

Figure S9. The impact of water on the catalytic performance of [Fe^{2+}/IGOPS/PP_3]. Total volume of H_2O =0.4 ml

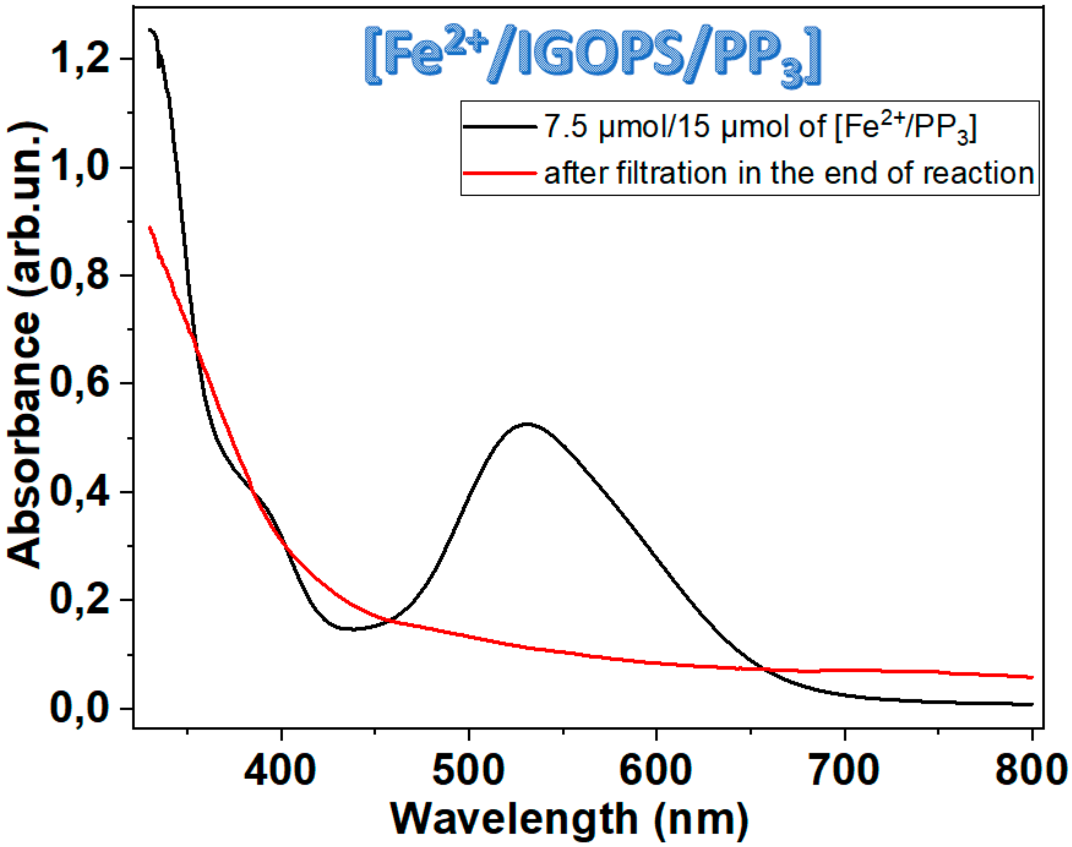

Figure S10. Leaching test of Fe^{II} after the end of reaction for the catalytic system $[Fe^{2+}/IGOPS/PP_3]$.

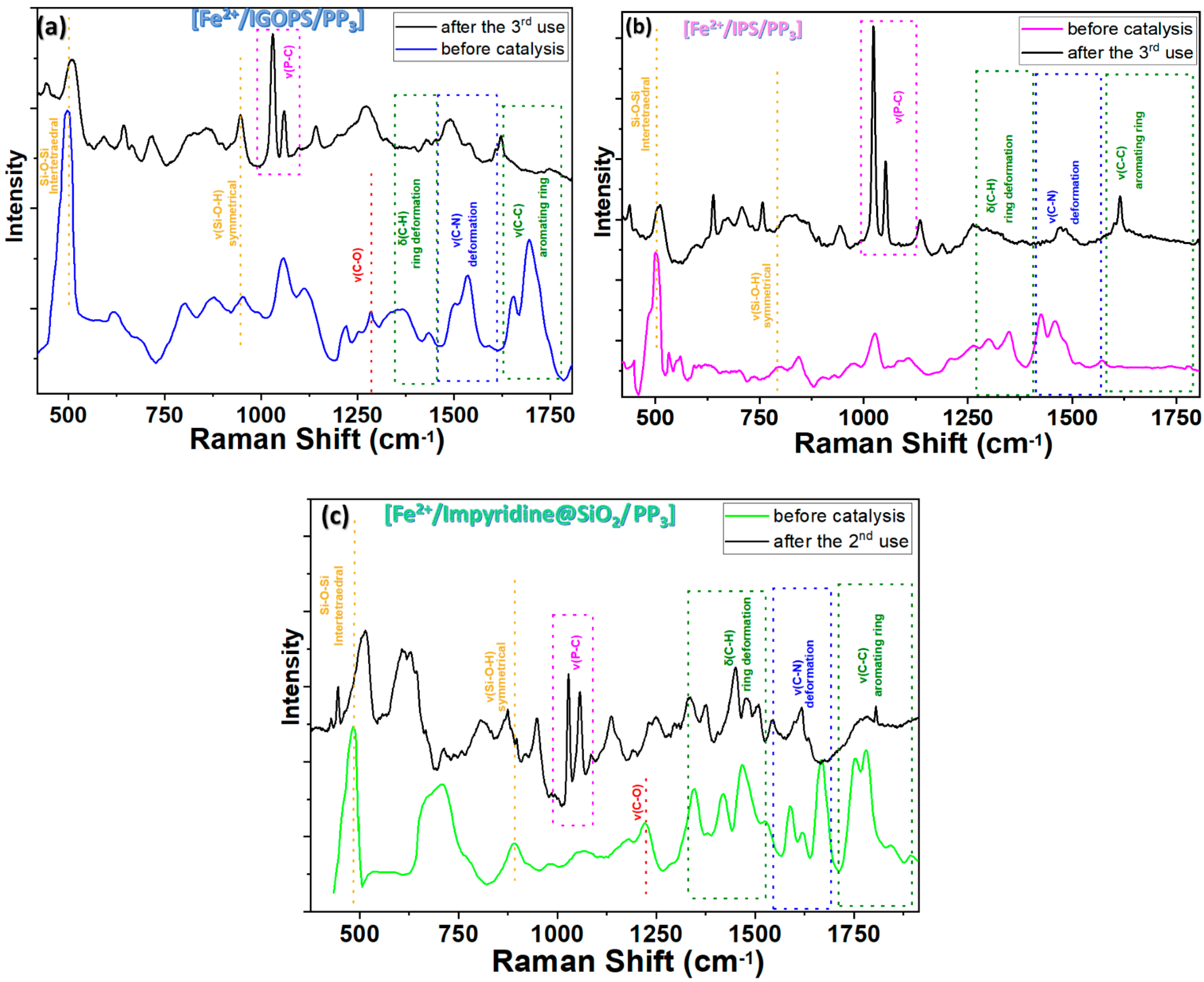

Figure S11. Raman spectra of (**a**) IGOPS (**b**) IPS (**c**) Impyridine@SiO_2 nanohybrids after reusing experiments

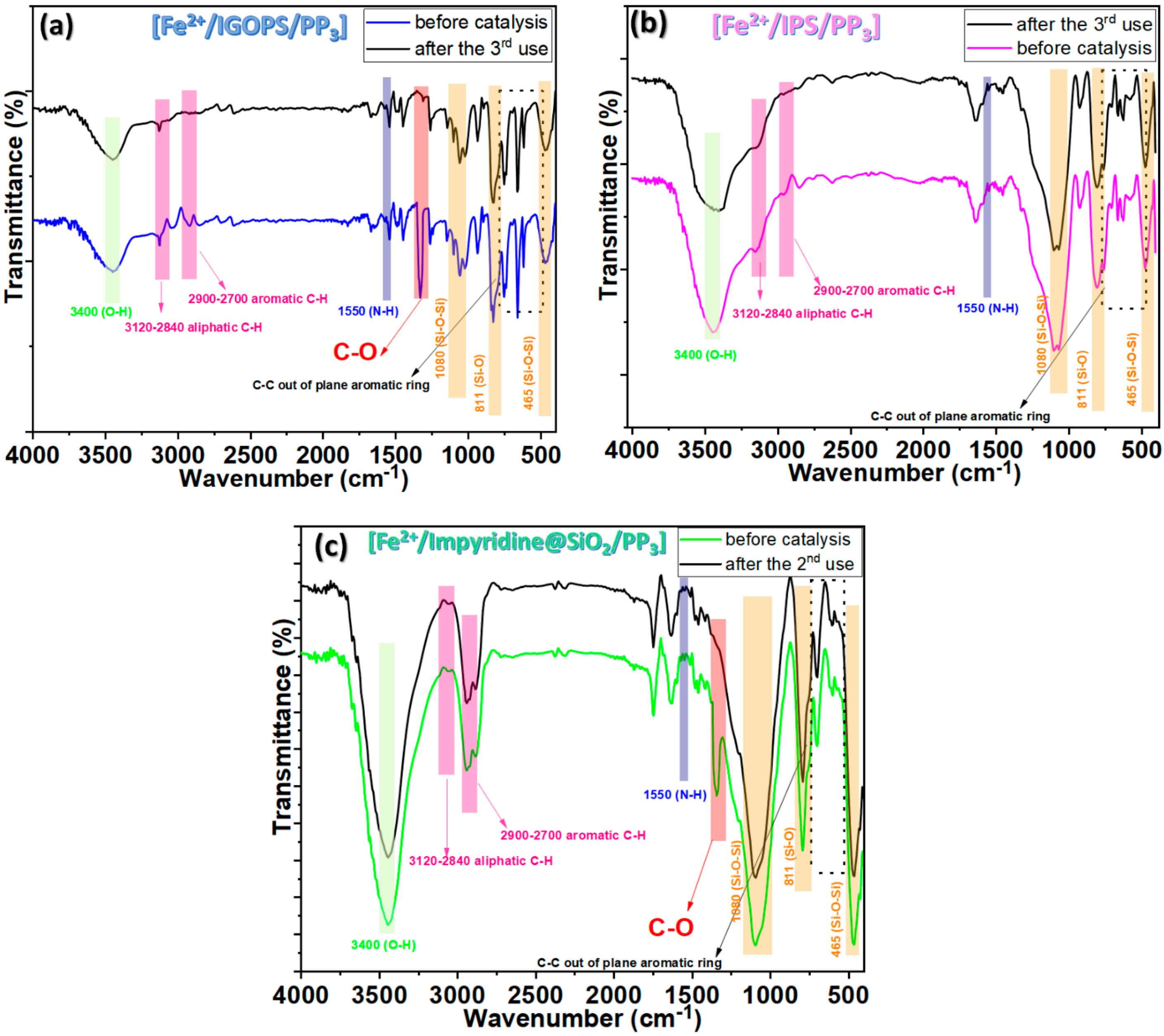

Figure S12. FT/IR of (a) IGOPS (b) IPS (c) Impyridine@SiO_2 nanohybrids after reusing experiments.
